# The effects of a neighbour and its identity on roots’ plastic growth

**DOI:** 10.1093/aobpla/plaf031

**Published:** 2025-06-24

**Authors:** Valentina Simonetti, Laura Ravazzolo, Benedetto Ruperti, Silvia Quaggiotti, Umberto Castiello

**Affiliations:** Department of General Psychology, University of Padova, Via Venezia 8, 35131 Padova, Italy; Department of Agronomy, Food, Natural Resources, Animals and Environment (DAFNAE), University of Padova, Viale delle università 16, 35020 Agripolis, Italy; Department of Agronomy, Food, Natural Resources, Animals and Environment (DAFNAE), University of Padova, Viale delle università 16, 35020 Agripolis, Italy; Department of Agronomy, Food, Natural Resources, Animals and Environment (DAFNAE), University of Padova, Viale delle università 16, 35020 Agripolis, Italy; Department of General Psychology, University of Padova, Via Venezia 8, 35131 Padova, Italy; Plants, Ecosystems & Climate

**Keywords:** roots, 3D movement, social growth

## Abstract

Plant responses to the presence of neighbours and social interactions between them have the potential to alter fundamental aspects of plants’ evolution, persistence, and coexistence. The present study employs a novel approach to investigate the three-dimensional movement of root tips in response to a neighbouring plant of the same or different species. We collected data from maize and pea plants in three experimental conditions: (i) individual condition, in which plants grew without neighbours; (ii) social growing condition with a conspecific neighbour, in which plants grew in the presence of another plant of the same species; and (iii) social growing with a heterospecific neighbour, in which plants grew in the presence of another plant of a different species. The results indicate that roots display a more pronounced ‘exploratory’ behaviour when growing under social conditions. For both maize and pea plants, a higher incidence of aggregative behaviour (primary root moving towards the neighbour) was observed when plants grew near a conspecific when compared with an heterospecific neighbour. According to our analyses, roots showing aggregative behaviour seem to detect the neighbouring root with a good level of geometrical precision as shown by the observed directional movement. We contend that this study provides for the first time quantitative information on the modulation of kinematic and oscillatory features of root movements, which are vital for a deeper understanding of plants’ below-ground interactions.

## Teaser

Plants interact with their neighbours below ground, and this study reveals that roots adjust their growth patterns based on both the presence and identity of nearby plants. Using a novel 3D imaging technique combined with kinematic analysis, we found that maize and pea roots modify their movement depending on whether the neighbouring plant is a conspecific or heterospecific. Roots exhibit more exploratory behaviour in the presence of a neighbour and are more likely to grow directionally toward conspecifics, demonstrating spatial awareness. This study provides quantitative evidence of how root kinematics and oscillatory features are modulated during below-ground interactions, offering new insights into plant behaviour and contributing to our understanding of root-driven coexistence, competition, and ecosystem dynamics.

## Introduction

Whether plants detect and respond to neighbouring plants is crucial to inform a complete understanding of how plants interact with one another. Plants’ interaction with neighbours involves various mechanisms that can be passive (e.g. resource depletion) and active (e.g. allelopathy, growth modifications). When investigating active responses to neighbours, there is evidence supporting the idea that plants can actively detect and distinguish their neighbours, and that they can actively respond to this presence by altering their growth or behaviour compared with growing alone (Bilas et al. 2021).

Plants respond to the presence of neighbouring plants by putting in place a variety of mechanisms to enhance their chances of thriving close to one another and that can be triggered by environmental cues (Schenk 2006, Pierik et al. 2013), such as light (Roig-Villanova and Martínez-García 2016), touch (Massa and Gilroy 2003, Markovic et al. 2016, Zhou et al. 2017 ), and chemical signalling (Biedrzycki et al. 2010, Karban et al. 2013, Semchenko et al. 2014, Kong et al. 2018, Yang et al. 2018).

By altering the placement of roots, plants implement sophisticated strategies that have important implications for their fitness such as nutrients and water intake (Cahill and McNickle 2011, Semchenko et al. 2014), allowing them to compete or cooperate depending on the nature of their neighbours (Brooker 2006, Bilas et al. 2021). For instance, the plant *Cakile edentula* var. *lacustris* shows greater root allocation when sharing a pot with a stranger than when sharing a pot with a kin (Bhatt et al. 2011), as previously hypothesized by Dudley and File (2007). This strategy is expected to increase the intake of resources at the expense of the neighbouring plant resulting in a form of competition. If the neighbour adopts the same strategy, it may lead to higher energy expenditure for growth, with a lower gain in resources, or it could result in the depletion of all the resources in a scenario known as the ‘tragedy of the commons’ (Gersani et al. 2001, Novoplansky 2009, Smy”ka and Herben 2017) as reported in different annual plants like soya bean (*Glycine max*, William’s variety; Gersani et al. 2001), beans (*Phaseolus varigaris*, var. Kenya; Maina et al. 2002), and peas (*Pisum sativum* L.; O’Brien et al. 2005). Cooperation among plants also appears to play an important role (Connell and Slatyer 1977, Weaver and Grime 1980, Bruno et al. 2003, Dudley 2015, Montazeaud et al. 2020) as far as kinship is concerned. For instance, it has been observed that the physical connection between plants, at least in some clones of *Fragaria chiloensis* L., can induce root segregation increasing plant performance (Holzapfel and Alpert 2003). Plants of *Ambrosia dumosa* decrease their rates of elongation in response to contact with roots of conspecific plants from the same population, but not so when they contact roots of conspecific plants from a different region (Mahall and Callaway 1996). From a more collective perspective, growing roots may exhibit coordinated behaviour that allows them to exploit the soil resources optimally. Active swarming (Baluška et al. 2010, Ciszak et al. 2012, Barlow and Fisahn 2013) could be considered a pattern of cooperative behaviour (Barlow and Fisahn 2013). According to Baluška et al. (2010), for an optimal exploitation of soil resources, plants developed complex and extended root systems that most of the time bring them to share the same space with conspecifics. Root swarming, as an expression of collective motion in general, can be a result of an evolutionarily conserved behavioural strategy that operates within the sphere of ecological interactions (Gómez et al. 2010).

Despite the importance of social interactions among plants, ecologists have only recently begun to explicitly study them through a behavioural lens (Gersani et al. 2001, Cahill and McNickle 2011, Mcnickle and Brown 2014, Belter and Cahill 2015). By using concepts derived from the field of behaviour, it is possible to draw upon a rich conceptual foundation to understand deterministic and plastic growth patterns in plants growing in social conditions (Belter and Cahill 2015). In their review, Cahill and McNickle (2011), referring to the overall structure of root architecture, identified three types of behaviour exhibited by the root system in response to the presence of neighbours: no response, avoidance, and aggregation. ‘No response’ behaviour indicates that plant root placement would be constant regardless of the presence or identity of a neighbour; ‘aggregation’ behaviour occurs if plants increase root growth in the vicinity of a neighbour; and ‘avoidance’ behaviour occurs if the plant shows undermixing of roots in the presence of a neighbour.

In this study, we capitalize on this behavioural taxonomy to explore the dynamic processes that determine where plants place their roots, focusing on the response of the root system to the presence of neighbouring plants. So far, studies investigating the spatial response of roots to neighbours, such as biomass allocation or directional growth, have mainly focused on static root features such as root mass, distribution, and morphology at a specific time point that usually occurs at the end of the experiment (Gersani et al. 2001, Falik et al. 2003, Hodge 2004, O’Brien et al. 2005, Gottlieb and Gruntman 2024). Little is known about kinematics features and their modulation over time as an effect of roots’ plastic growth in response to neighbouring plants. In plants, kinematic modulation and plastic growth can be associated to the final goal driving the movement (Bonato et al. 2023). A variety of studies on climbing plants have uncovered the anticipatory modulation of movement kinematics for reaching a final goal (e.g. a potential support; Guerra et al. 2019, Ceccarini et al. 2020) or in response to the presence of a neighbouring plant (Bonato et al. 2023, 2024). Because of the hidden nature of roots, the study of plastic growth and kinematic modulation of this organ is complex and poorly studied. Here, we attempt to fill this gap by examining whether root kinematics and its modulation over time is influenced by the presence of another plant of the same or different species sharing the same substrate by using the analysis of root movements in three-dimensional (3D) space. We also investigated whether roots exhibit goal-oriented directional movements in response to a neighbour.

## Materials and methods

### Sample description

We considered 37 maize (*Zea mays* L. (B73)) and 20 pea (*P. sativum* L., var. saccharatum) plants. Among these, we included data collected from 19 maize (*Z. mays* L. (B73)) reported in a previous study (Simonetti et al. 2024).

### Experimental conditions

We considered three experimental conditions, one with the plant growing alone (termed ‘individual’ condition) and the other two including a pair of plants growing together sharing the same substrate (termed ‘social’ condition):

- individual condition, in which plants grew without neighbours (*Z. mays* L. (B73)) (*n* = 19);
- social growing with conspecific, in which plants grew in the presence of another plant from the same species (*Z. mays* L. (B73) vs. *Z. mays* L. (B73) and *P. sativum* L., var. saccharatum vs. *P. sativum* L., var. saccharatum) (for maize *n* = 10, for pea *n* = 12);
- social growing with heterospecific, in which plants grew in the presence of another plant from a different species (*Z. mays* L. (B73) vs. *P. sativum* L., var. saccharatum) (for maize *n* = 8, for pea *n* = 8).

### Germination and growth conditions

**Figure 1.**
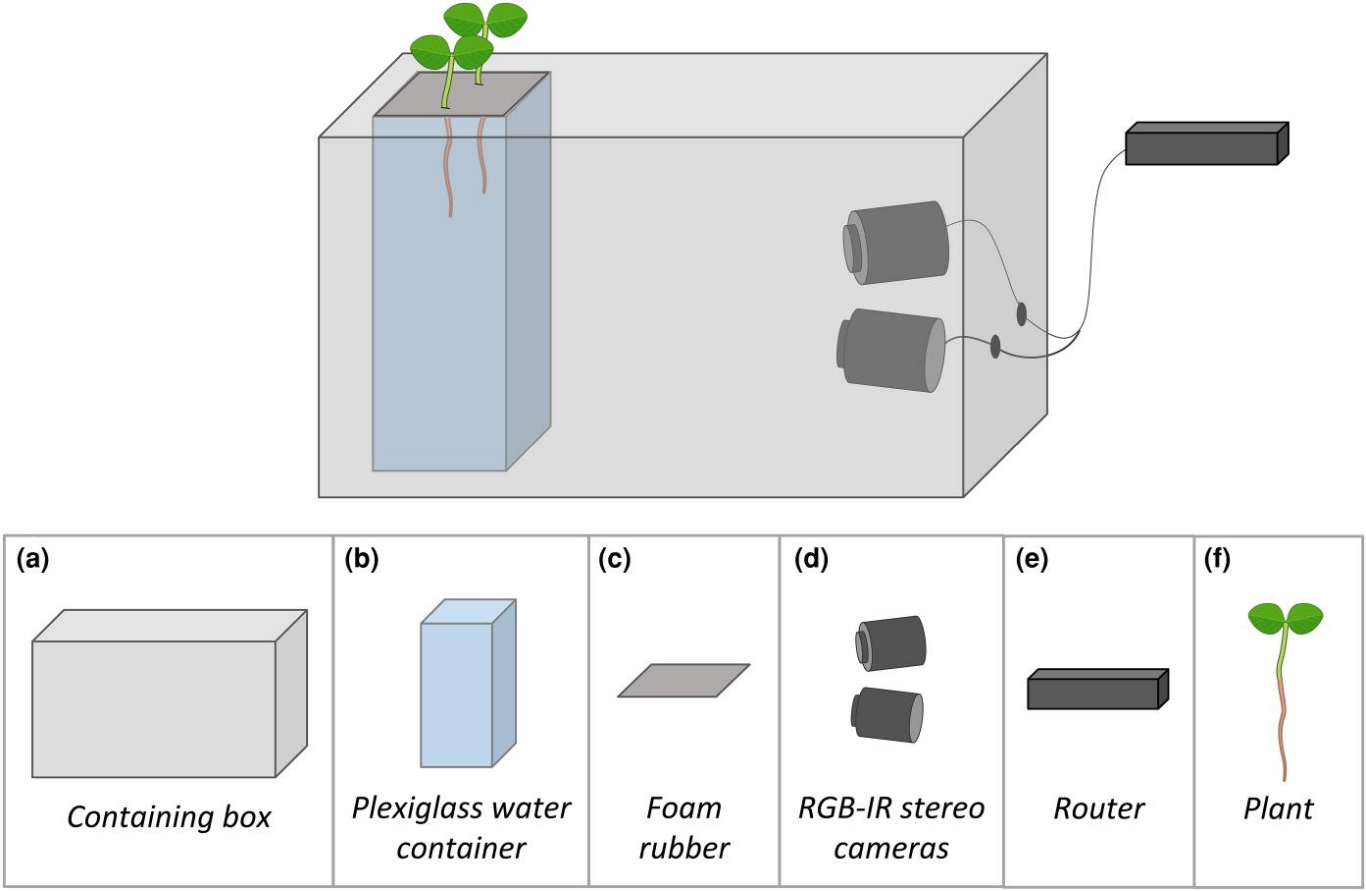
Top: system configuration. The roots grow in a transparent plexiglass container filled with a water nutrient solution while a pair of stereo cameras take a picture of it every 3 min. Both the container and the stereo cameras are placed inside a box that ensures roots grow in complete darkness. The whole system is placed inside a growing chamber under a controlled light condition. Bottom: components of the system. (a) Containing box: carton box 60 cm × 40 cm × 40 cm. (b) Plexiglass water container: plexiglass container 10 cm × 10 cm × 30 cm filled with water nutrient solution (modified Hoagland nutrient solution; Quaggiotti et al. 2003). (c) Foam rubber: foam rubber rectangle (11 cm × 11 cm × 1.5 cm) used to host and stabilize the seed. (d) RGB-IR stereo cameras: IP 2.1 Mpx outdoor varifocal IR 1080 P. Each camera is wired with ethernet cables to a router. (e) Router: D-link Dsr-250n connected via Wi-Fi to a PC. (f) Plant: either *Z. mays* L. (B73) or *P. sativum* L. (var. saccharatum). Adapted from Simonetti et al., (2024), CC BY 4.0.

Seeds were germinated on filter paper adopting the same seed orientation, with the extremity where the root emerges facing down. They were kept in the dark at a constant temperature of 25°C. Four-day-old seedlings were measured by positioning the seedling on a horizontal surface and measuring the root length from the lower tip of the seed to the root tip. We selected seedlings with an average root length of 4 cm ± 1 cm. The plants grew in hydroponic culture inside a transparent acrylic glass container (PLEXX, Piazzola sul Brenta PD; see Fig. 1) filled with a nutrient solution (a modified Hoagland nutrient solution; Quaggiotti et al. 2003) with a day/night cycle of 14/10 h. Data collection for each plant was conducted for 7 days. To stabilize the seedling, the seed was placed in a 1 cm-thick foam rubber support with the root inside the solution and the shoots outside (Fig. 1). Plants were positioned at a relative distance of 5 cm from the neighbouring plant. We placed two plants in each container. To maximize the gas exchange with the atmosphere, four symmetrical holes with a diameter of 7 mm were placed in the foam rubber support. For each experiment, plants were randomly assigned to the experimental condition. We used four identical growing chambers, each containing one acquisition system shown in Fig. 1. The chambers guarantee that light and temperature conditions are kept constant across plants and experimental conditions. As an additional environmental control, we placed a digital thermo-hygrometer (Govee Bluetooth Hygrometer Thermometer H5075) inside each growing chamber to constantly monitor temperature and humidity during the experiment. Additionally, the experimental combinations were counterbalanced across chambers to avoid chamber-specific bias.

### Data acquisition

The data acquisition system used for this research is exhaustively described in Simonetti et al. (2024). In brief, a pair of RGB-IR stereo cameras (IP 2.1 Mpx outdoor varifocal IR 1080 P.) were used to take pictures of the root every 3 min (0.0056 Hz) for 7 days. Both the container for a hydroponic culture and the stereo cameras were kept inside a box to maintain the root in complete darkness while the upper part of the plant grows in normal light conditions. Following the same protocol as in Simonetti et al. (2024), we extracted the 3D trajectory of the primary root tips along the time acquisition of the experiment, triangulating their position from the time lapses obtained by the stereo cameras. We processed the acquired time lapses using the SPROUTS software (Ab.Acus srl Milan, Italy; Simonetti et al. 2021).

### Extracted features

We processed the 3D trajectories of the primary root tips to extract the features we used for the analyses. The features extracted from the root tip trajectories are described below.

#### Kinematic features: distance, velocity, and acceleration

We first calculated the main kinematic quantities between all of the consecutive points of the root tip 3D trajectory. Then, we used the time series obtained for distance, velocity, and acceleration to extract descriptive statistics (mean, median, maximum, and minimum) for each plant for the overall acquisition.

#### Segmentation angles

Segmentation angles are the angles between consecutive segments of a trajectory.

Given three consecutive points in 3D space, $\left(x_{i-1},y_{i-1,\;}z_{i-1}\right)$, $\left(x_{i},y_{i,\;}z_{i}\right)$, and $\left(x_{i+1},y_{i+1,\;}z_{i+1}\right)$, the angle *θ* between the vectors formed by these points is calculated using the dot product formula.

The vectors are as follows:*θ*


(1) $$\begin{array}{c} v_{\mathrm{prev}}=(x_{i}-x_{i-1},y_{i}-y_{i-1},z_{i}-z_{i-1}) \end{array}$$

(2) $$\begin{array}{c} v_{\mathrm{next}}=(x_{i+1}-x_{i},y_{i+1}-y_{i},z_{i+1}-z_{i}) \end{array}$$

The angle *θ* between these vectors is calculated as follows:


(3) $$\begin{array}{c} \theta =\arccos\;(\cos\;(\theta )) \end{array}$$

where


(4) $$\begin{array}{c} \cos\;(\theta )=\frac{v_{\mathrm{prev}}\cdot v_{\mathrm{next}}}{\left|\left|v_{\mathrm{prev}}\right||\;|\left|v_{\mathrm{next}}\right|\right|}\; \end{array}$$

We calculated segmentation angles for all consecutive points of the root tip trajectory, which reflect how sharply the 3D trajectory changes direction at each point. Smaller angles indicate sharper turns, whereas larger angles indicate smoother transitions. We used the time series of segmentation angle values to extract descriptive statistics (mean, median, maximum, and minimum) for the overall acquisition.

#### Primary root growth rate

We computed the root length growth rate as both absolute and relative root growth rate as in Simonetti et al. (2024).

#### Average hourly tip velocity

We assessed the average tip velocity as the average distance travelled by the root tip per hour (Simonetti et al. 2024).

#### Nutation amplitude

We calculated nutation amplitude as the displacement from the main component of growth. Then, we calculated the time series of nutation amplitudes to calculate descriptive statistics (mean, median, maximum, and minimum) for the overall acquisition (Simonetti et al. 2024).

#### Main period of nutation

We calculated the main period of nutation by applying a discrete Fourier transform by means of the fast Fourier transform algorithm (Cooley and Tukey 1965) to the time series of nutation amplitude. We computed the main period of nutation as the period corresponding to the frequency in the power spectrum where the maximum amplitude value was detected (Simonetti et al. 2024).

#### Spectral entropy

Spectral entropy is a measure of the complexity or randomness of a signal in the frequency domain, and it provides insights into the distribution of power across different frequency components of the signal (Rezek and Roberts 1998). Spectral entropy is derived from the power spectral density (PSD) of a signal. Given a signal s(t) sampled at a frequency $f_{s}$, the PSD $P_{ss}(f)$ represents how the power of the signal is distributed over different frequency components. Spectral entropy *H* is defined as follows:


(5) $$\begin{array}{c} H=\;-\sum_{i}P_{i}\log_{2}(P_{i})\; \end{array}$$

where $P_{i}$ is the normalized power at the *i*th frequency component.

In our case, the signal s(t) was the array of Euclidean distances on the *XY* plane between each sample on the root tip trajectory and the root main component of growth. PSD was estimated using the Welch method (Welch 1967). The spectral entropy calculated for the trajectory of the root tip provided a measure of the complexity of the root tip’s oscillatory movement in the frequency domain. Following the definition of entropy (Shannon 1948), a high spectral entropy indicates a wide and uniform distribution of power across frequencies, suggesting a complex or random signal in terms of frequency content. Conversely, a low spectral entropy indicates that the power is concentrated in a few frequencies, suggesting a more regular or predictable signal.

#### Total variation of curvature

Curvature is a measure of how sharply a curve bends at a given point. Curvature provides a local measure of how sharply a curve bends (Gray et al. 2017). For a curve parameterized by r(t) in 3D space, where r(t)=(x(t),y(t),z(t)), the curvature k(t) at a× point is given by


(6) $$\begin{array}{c} k(t)=\frac{\left|r^{\prime }(t)\times r^{\prime \prime }(t)\right|}{{\left|r^{\prime }(t)\right|}^{3}} \end{array}$$

where $r^{\prime }(t)$ and $r^{\prime \prime }(t)$ are the first and second derivatives of r(t) with respect to t and is the cross product. The numerator represents the magnitude of the binormal vector, which indicates the direction of the curve’s deviation from a straight path, whereas the denominator normalizes this value based on the speed of the trajectory.

We calculated the total variation of curvature for the root tip trajectory r(t) over the interval of the experiment [a,b] as follows:


(7) $$\begin{array}{c} \mathrm{TVC}(k,[a,b])=\overset{n}{\underset{i=1}{\sum }}\;|k(t_{i})-k(t_{i-1})| \end{array}$$

The value obtained summarizes the overall change in curvature along the trajectory. A high total variation indicates that the root trajectory undergoes significant changes in bending, whereas a low total variation suggests a more uniform bending behaviour.

#### Tortuosity

Tortuosity quantifies how much the root trajectory deviates from a straight line, and we calculated it as the ratio of the total length of the root trajectory over the 3D distance between the start and end points of the trajectory.


(8) $$\begin{array}{c} \mathrm{Tortuosity}=\;\frac{\mathrm{Overall}\;\mathrm{trajectory}\;\mathrm{length}}{\mathrm{Euclidean}\;\mathrm{distance}\;(\;p(0),p(\mathrm{end}))} \end{array}$$

A higher tortuosity value indicates a more convoluted root tip trajectory.

### Data analysis

**Figure 2.**
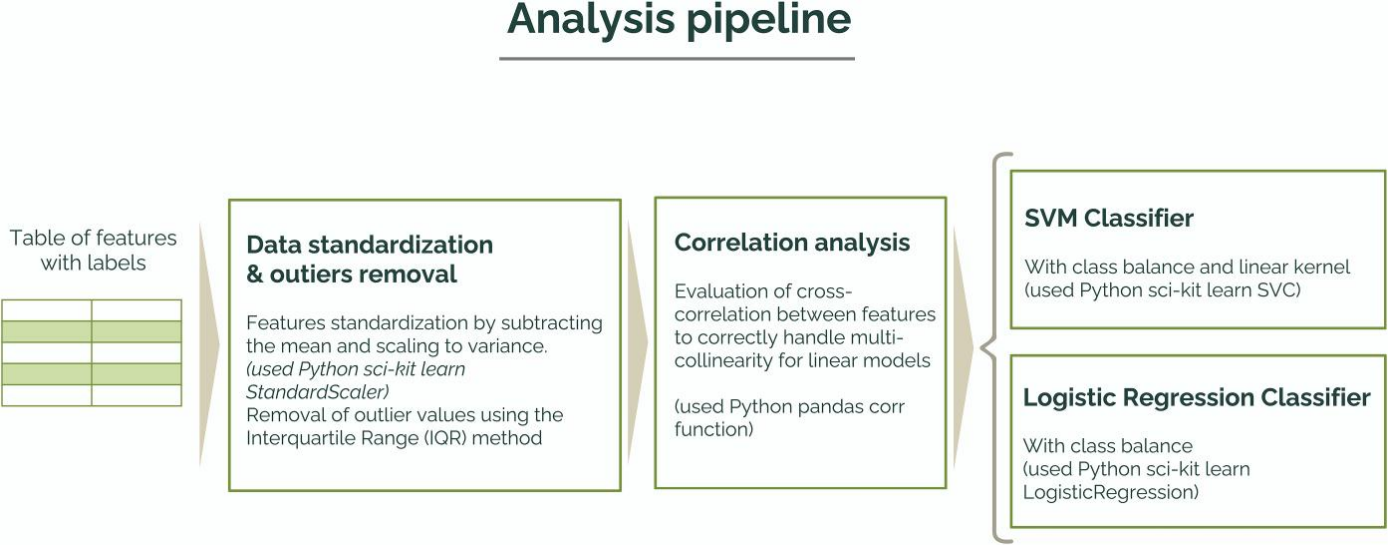
Analysis pipeline used to build the models to classify the target conditions individual vs. social. The table of features had 37 rows (19 rows for individual condition and 18 rows for social condition). The columns of the table were the extracted features and the labels (target condition). The data were first standardized, and outlier values were removed to balance their contribution to the final classification. A correlation analysis was then performed to assess cross-correlation between variables, thus identifying those couples of variables showing cross-correlation >0.7 and retaining only one variable from each pair. This selection was performed to avoid issues related to multicollinearity. The resulting table of features was then used to train and test two classifiers: an SVM with a linear kernel and an LR.

We performed the first analysis to compare maize plants grown under an individual condition vs. maize plants growing in a social condition. We used a machine learning–based approach to evaluate whether the features extracted from root movement could be used to distinguish among the two conditions of growth. We implemented two computational models for classification that were trained and tested using the features extracted from the two conditions in order to classify the condition itself (individual vs. social). The final goal was not to build models able to classify these two conditions based on the features extracted, but rather to evaluate whether these models could distinguish between the two conditions on a multivariate basis. With this approach, we could leverage on the feature importance analysis provided by the classification models to obtain a functional interpretation of the classification results, in order to better explain the differences between the two conditions of growth. Given the sample size, we used linear models to perform the classification as they are less prone to overfitting compared with more complex models: a logistic regression (LR) model and a support vector machine (SVM) with a linear kernel. Both the models used are included in the Python package Scikit-learn (Pedregosa et al. 2011). We standardized all the features in terms of mean and variance in order to correctly balance their contribution to the final classification. To properly address the issue of multicollinearity in linear models, we calculated a cross-correlation matrix for all the features extracted, excluding the couple of features that showed a cross-correlation absolute value >0.7. After this process, the features selected to train and test the classification models were as follows: (i) ‘mean speed’, (ii) ‘minimum speed’, (iii) ‘tortuosity’, (iv) ‘mean nutation amplitude’, (v) ‘period of main nutation’, (vi) ‘total variation of curvature’, (vii) ‘average relative daily growth rate’, (viii) ‘amplitude of the main frequency in the power spectrum’, (ix) ‘spectral entropy’, and (x) ‘mean segmentation angle’. For both models, we used a five-fold cross-validation to assure that the final classification outcomes were not dependent on a particular subset of the data. Figure 2 shows the complete analysis pipeline.

In the following, we investigated the effects of the identity of the neighbouring plant (conspecific or heterospecific) on root growth, performing an analysis of root movements in 3D space. We also investigated whether roots exhibit oriented directional movements in response to a neighbour. To this end, the movement of the primary root was classified on the basis of three possible behaviours:

- ‘Neutral’ behaviour: when the root of the plant did not exhibit clear directional growth either towards the root of the other plant or in the opposite direction
- ‘Avoidant’ behaviour: when the root of the plant grew in the opposite direction of the root of the other plant
- ‘Aggregative’ behaviour: when the root of the plant grew towards the root of the other plant

### Classification procedure

We performed the classification of root growth behaviour (neutral, avoidant, and aggregative) computationally, evaluating the movement of the root tip in space and time. When the root initially showed a specific behaviour and then it switched to another one, we only considered the first behaviour. We outline the details of the algorithm used below:

Let $r_{\;p0}=(r_{\;p0x},r_{\;p0z})$ be the initial point of the root tip trajectory of the plant, where $r_{\;p0x}$ and $r_{\;p0z}$ are the *x* and *z* coordinates, respectively.

Let $r_{n0}=(r_{n0x},r_{n0z})$ be the initial point of the roo$r_{n0z}$t tip trajectory of the neighbour plant, where $r_{n0x}$ and are the *x* and *z* coordinates, respectively.

We calculated the vector between the root tip of the plant and the root tip of the neighbour as follows:


(9) $$\begin{array}{c} \vec{PN}=\;r_{\;p0}-r_{n0} \end{array}$$

Then, for every hour of the experiment, we calculated the vector of the displacement of the root growth main component (calculated as in Simonetti et al. 2024) on the *XZ* plane as


(10) $$\begin{array}{c} \vec{PP_{h}}(t)=\;r_{p}(t)-r_{p}(t+1\;h) \end{array}$$

Then, we calculated the growth angle $\theta_{gh}$ between $\vec{PN}$ and $\vec{PP_{h}}$ for every hour of the experiment as


(11) $$\begin{array}{c} \theta_{gh}=\arccos\;(\cos\;(\theta_{gh})) \end{array}$$

where


(12) $$\begin{array}{c} \cos\;(\theta_{gh})=\frac{\;\vec{PN}\cdot \vec{PP_{h}}}{\left|\left|\vec{PN}\;\right||\;|\left|\vec{PP_{h}}\right|\right|} \end{array}$$

**Figure 3.**
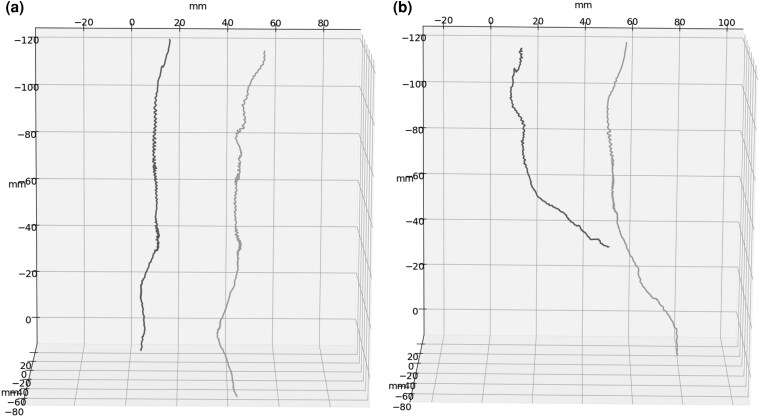
(a) Sample trajectories of two plants showing neutral behaviour. (b) Sample trajectories of two plants showing aggregative behaviour (left) and avoidant behaviour (right).

We obtained the absolute value of the hourly growth angle $\left|\theta_{gh}\right|$ towards the neighbour plant for every hour of the experiment. The value of $\left|\theta_{gh}\right|$ can range between 0° and 180°, where 0° means that the root is growing exactly towards the root of the neighbour, and 180° means that the root is growing in the exact opposite direction. When $\theta_{gh}$ has a value <90°, the root tip movement is considered towards the neighbour; however, when $\theta_{gh}$ has a value >90°, the root tip movement is considered away from the neighbour. Together with movement direction, we also calculated the spatial displacement of the root tip in the *XZ* plane as the module of the vector of the hourly displacement of the root growth main component $\vec{|PP_{h}}(t)|$. We considered a movement towards the neighbour as positive displacement, whereas movement in the opposite direction was classified as negative displacement. If the cumulate value of the displacement exceeded 1.2 cm (one-fourth of the distance between the two plants), we classified the behaviour as aggregative; if the cumulate value of the displacement was found to be <−1.2 cm, we classified the behaviour as avoidant; and if the cumulate value of the displacement was never >1.2 cm or <−1.2 cm, we classified the behaviour on the basis of the highest incidence along the experiment. If none of the behaviours showed an incidence higher than the other for >35% of the total time, we classified the behaviour as neutral. Figure 3 shows some examples of trajectories for the three different behaviours (see also sample time-lapse videos in Supplementary Data). For the analysis on directional movements, we selected the plants with a primary root behaviour classified as aggregative. We extracted the global direction of root growth and calculated the angle between the global direction of growth and the vector connecting the two plants. We describe the mathematical procedure as follows.

Let $r_{\;p0}$, $r_{n0}$, and $\vec{PN}\;$ be as described above.

Let $r_{\mathrm{pend}}=(r_{\mathrm{pend}x},r_{\mathrm{pend}z})$ be the point of the root tip trajectory of the plant when a behaviour is identified, where and $r_{\mathrm{pend}z}$ are the *x* and *z* coordinates, respe$r_{\mathrm{pend}x}$ctively.

We then calculated the global vector of the displacement of the root growth main component on the *XY* plane as


(13) $$\begin{array}{c} \vec{P_{0}P_{\mathrm{end}}}\;=\;r_{\;p0}-r_{\mathrm{pend}} \end{array}$$

In the following, we calculated the growth angle $\theta_{g}$ between $\vec{PN}$ and $\vec{P_{0}P_{\mathrm{end}}}$ as


(14) $$\begin{array}{c} \theta_{g}=\arccos\;(\cos\;(\theta_{g})) \end{array}$$

where


(15) $$\begin{array}{c} \cos\;(\theta_{g})=\frac{\;\vec{PN}\cdot \vec{P_{0}P_{\mathrm{end}}}}{\left|\left|\vec{PN}\;\right||\;|\left|\vec{P_{0}P_{\mathrm{end}}}\right|\right|} \end{array}$$

**Figure 4.**
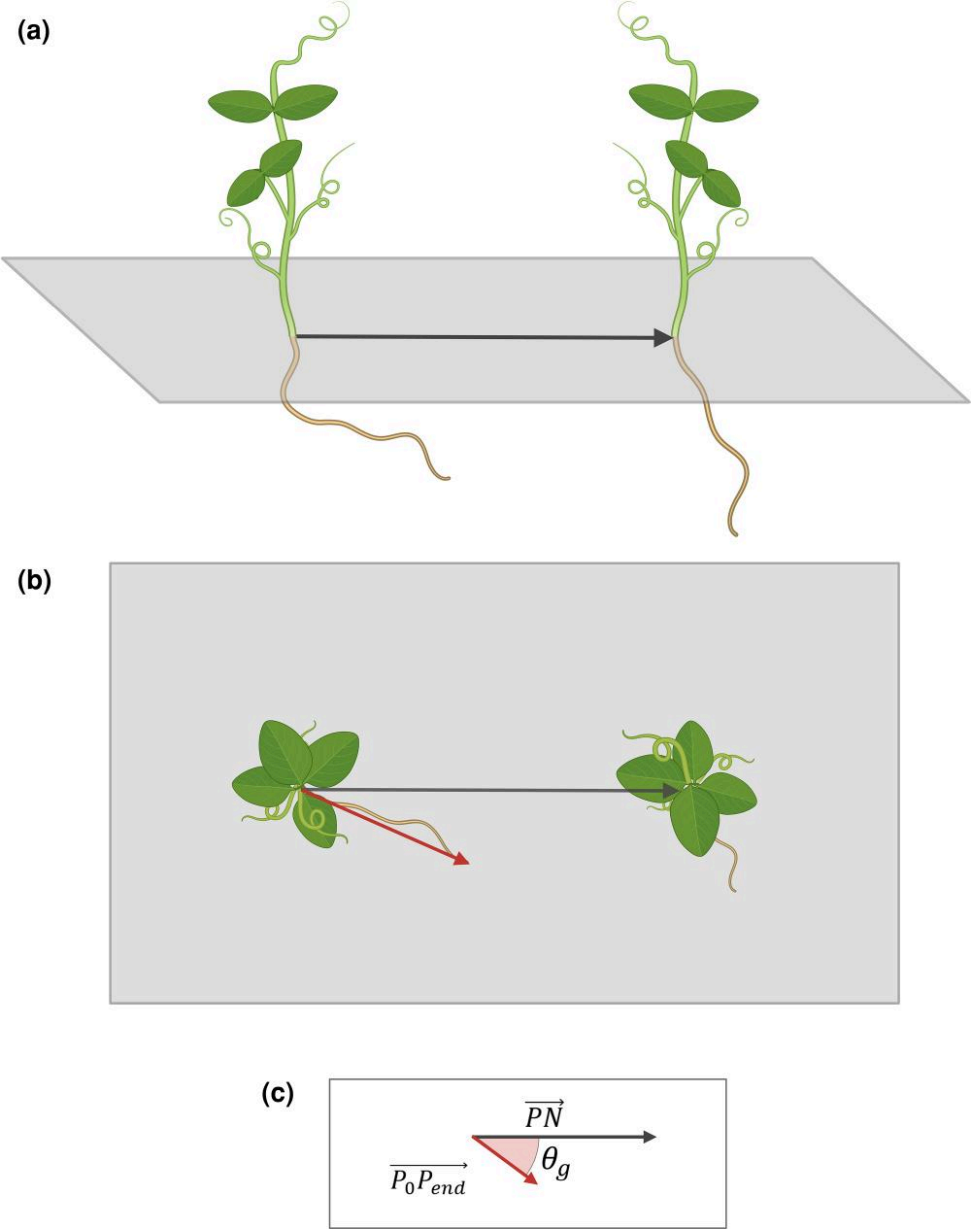
Graphical representation of the extraction of root growth angle $\theta_{g}$. (a) Side view of the experimental setup, where two plants grow together in social condition; the black arrow represents the vector between the root tip of the plant and the root tip of the neighbour at the beginning of the experiment ($\vec{PN}$). (b) Top view of the experimental setup; the black arrow always represents the vector between the root tip of the plant and the root tip of the neighbour at the beginning of the experiment ($\vec{PN}$), whereas the red arrow represents the vector of root growth from the beginning of the experiment to the end of the experiment (or when the identified behaviour changes) ($\vec{P_{0}P_{\mathrm{end}}}$). (c) Geometrical representation of the extraction of root growth angle $\theta_{g}$ as the angle between $\vec{PN}$ and $\vec{P_{0}P_{\mathrm{end}}}$.

The value of $\theta_{g}$ can range between −180° and 180°, where a value around 0° means that the root is growing exactly towards the root of the neighbour, and a value around ±180° means that the root is growing in the exact opposite direction. Figure 4 shows a graphical representation of the extraction of the root growth angle $\theta_{g}$.

## Results

### Individual vs. social growing

**Figure 5.**
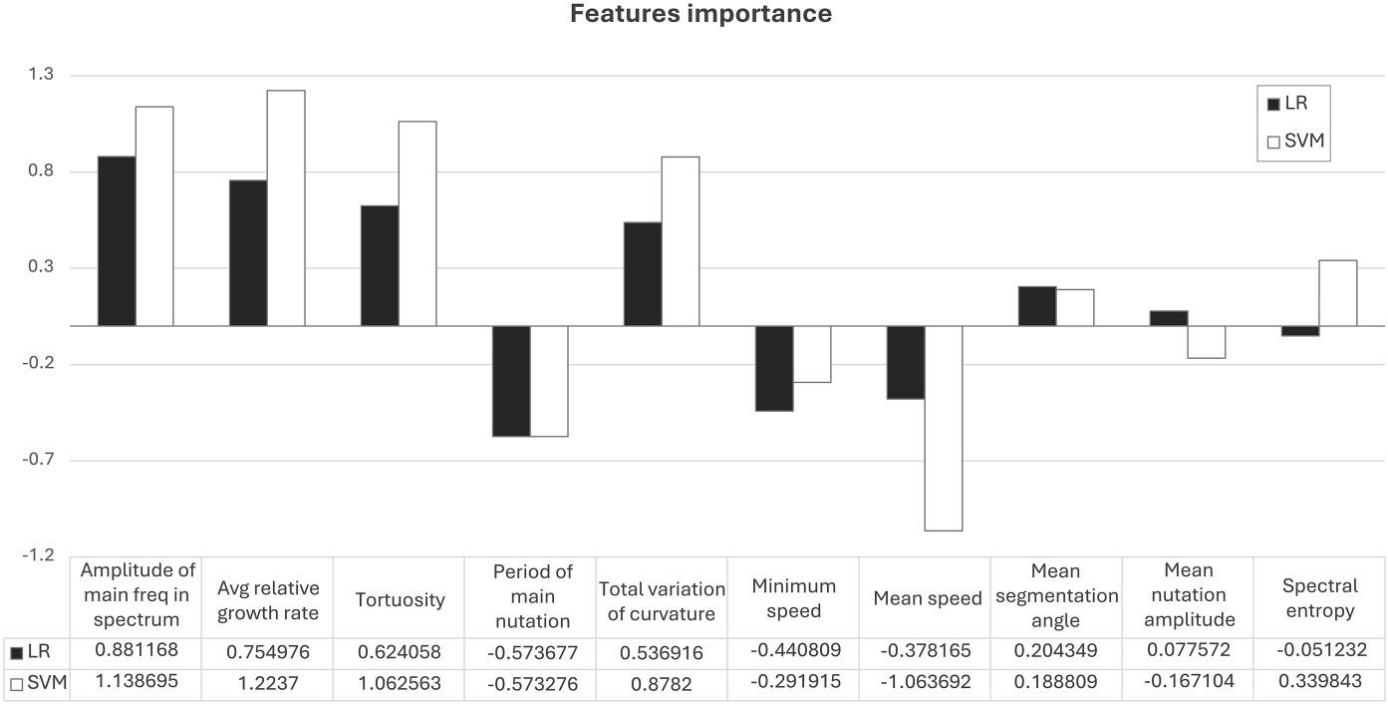
Results of the feature importance analysis. At the top: the graphical trend of the values of the coefficients. At the bottom: the values of the coefficients for all features for both LR and SVM classifiers.

Both the LR and the SVM classifiers showed good performance in distinguishing the two growing conditions (individual vs. social). This signifies that root growth is influenced by the presence of the neighbouring plant as evident in the modulation of the growth parameters investigated. Figure 5 shows the values of the coefficients obtained for both LR and SVM classifiers. LR reported a classification accuracy of 75% (SD = ±22%), whereas the SVM reported a classification accuracy of 89% (SD = ±11%). For both models, including the minimum range of standard deviation, we obtained a classification accuracy >50%, implying that, for our dataset, the two conditions can be discriminated by the trained algorithms. To provide a functional interpretation of the differences between the two conditions, we performed a feature importance analysis. For both LR and SVM classifiers, we analysed the coefficients of the models to collect insights into the degree and type of influence (positive or negative) that each feature has on the prediction. After analysing the values of the coefficients, we observed that the two models show a similar trend of features importance.

The agreement between the models provides evidence of the robustness of the overall data management process and classification results. From the identification of the most important features, we can provide a qualitative interpretation of the differences between the two conditions (individual vs. social). The features showing higher importance for both models (Fig. 5) are (i) ‘amplitude of the main frequency in the power spectrum’, (ii) ‘average relative daily growth rate’, (iii) ‘tortuosity’, and (iv) ‘period of main nutation’ (with a negative coefficient).

From this, we could infer that the root of a maize plant put in a social growing condition, when compared with the individual condition, exhibits the following:

1. More and faster nutations: the positive coefficient of the ‘amplitude of the main frequency in the power spectrum’ indicates a higher power, in the frequency domain, for the main frequency of nutation, whereas the negative coefficient of the ‘period of main nutation amplitude’ means a reduced nutation period for the social condition.
2. More complex curvature: this is evidenced by the positive coefficient for the ‘tortuosity’.
3. Faster relative growth rate: indicated by the positive coefficient of the ‘average relative growth rate’ feature.

**Figure 6.**
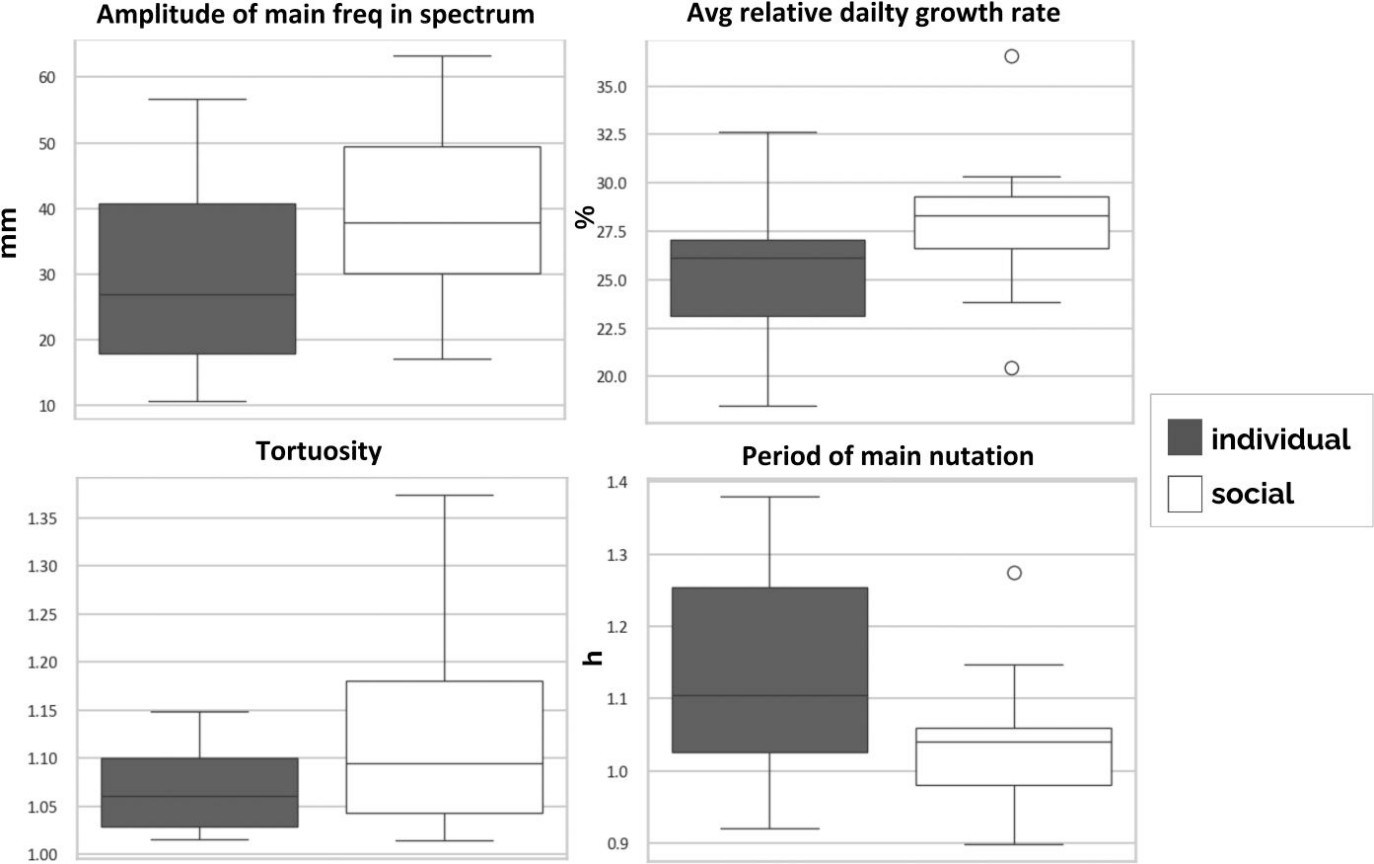
Boxplots of the distributions of values for the four features showing higher importance in classifying the individual vs. the social condition.

Investigating the distributions of the values for these variables, their trends confirm the qualitative interpretation of the results (Fig. 6).

### Social growing: roots’ behaviours and directional movements

**Figure 7.**
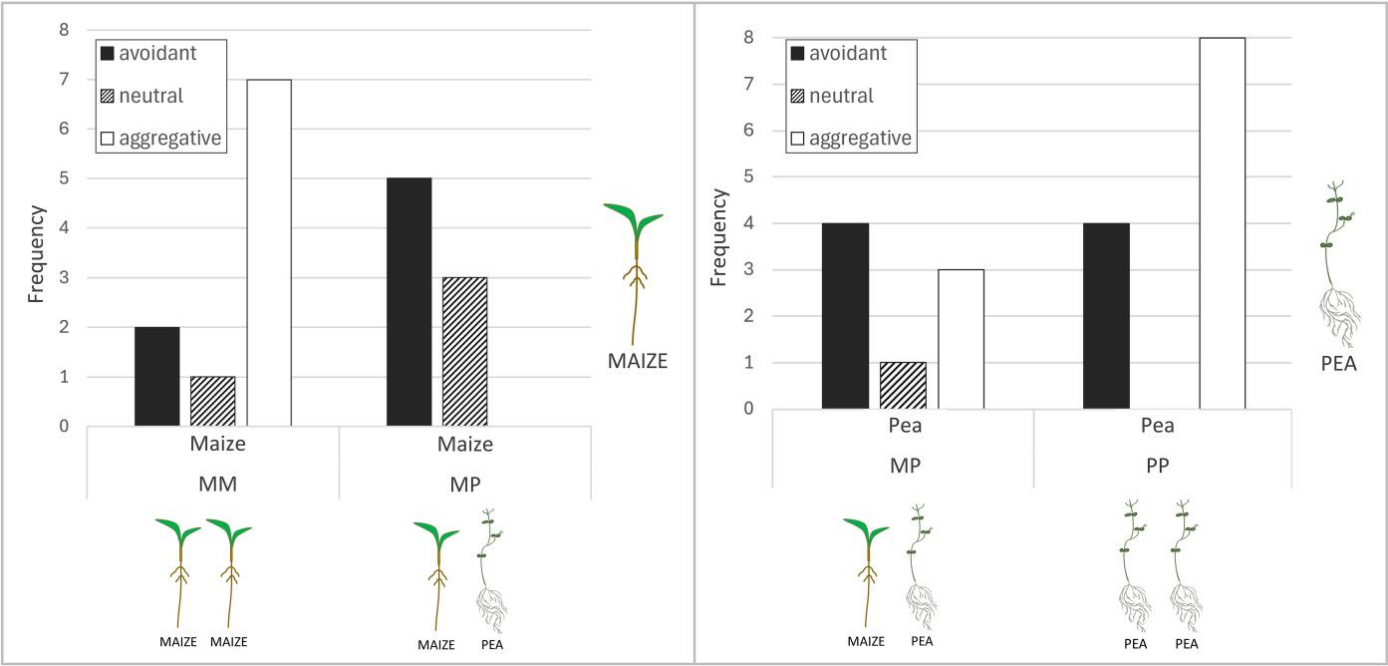
Bar plot of the distribution of behaviours in primary root for the study plants. On the left: distribution of behaviours for maize root growing with another maize or with a pea. On the right: distribution of behaviours for pea root growing with another pea or a maize.

We evaluated the incidence of the three possible behaviours exhibited by the primary root (neutral, avoidant, and aggregative) for the plants growing in a social growing condition to assess whether the species of the neighbouring plant was affecting root growth. Both for maize and pea plants, a higher incidence of aggregative behaviour was observed when the plants grew next to a conspecific rather than a heterospecific. Figure 7 shows these results by depicting the incidence of the three possible behaviours, in maize and for pea plants. When growing with a heterospecific, maize plants showed only avoidant and neutral behaviour, whereas pea plants showed mainly avoidant and aggregative behaviour in both configurations.

To test whether the frequency of aggregative behaviour was significantly higher when growing with a conspecific compared with a heterospecific, we first aggregated the data by growth condition (with a conspecific/with a heterospecific) and by observed behaviour (aggregative/non-aggregative). Table 1 shows the contingency table obtained.

**Table 1.** Contingency table showing the frequency of aggregative and non-aggregative behaviour under the two growth conditions: with a conspecific and with a heterospecific.

	Behaviour		
Condition	Aggregative	Non-aggregative	Total
Growth with conspecific	15	7	22
Growth with heterospecific	3	13	16
Total	18	20	38

On these data we run a χ^2^ test with Yates’ correction for continuity obtaining a test statistic of 7.204 with a *P*-value of .007, indicating a statistically significant association (*P* < 0.01) between growth condition and aggregative behaviour.

**Figure 8.**
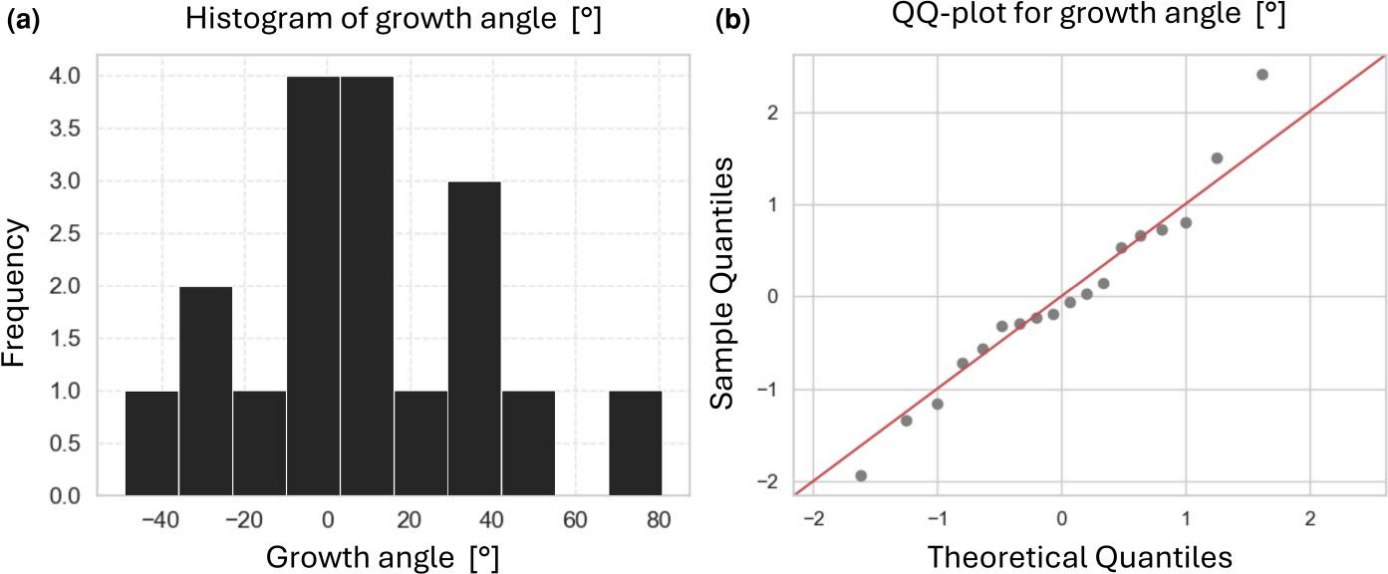
(a) Histogram of the values of root growth angle $\theta_{g}$ for all the plants showing aggregative behaviour. (b) QQ-plot of the same values of root growth angle $\theta_{g}$ against the values for a normal distribution.

For those cases in which the root showed aggregative behaviour, we investigated whether root growth was oriented towards the other plant, thus exhibiting a directional growth. We exploited our novel technology to investigate whether the aggregative movement had a level of geometrical precision through the evaluation of the 3D trajectory. Directionality of growth implies that the neighbouring plant becomes the spatial goal of the root movement in the environment. We selected all the plants exhibiting aggregative behaviour and we evaluated whether the root targets geometrically the other plant. To do this, we calculated the growth angle $\theta_{g}$ for all the plants and then extracted the histogram of the distribution of $\theta_{g}$ values and the QQ plot (quantile–quantile plot), to visually investigate the shape of the distribution and its comparison with a normal distribution (Fig. 8).

**Figure 9.**
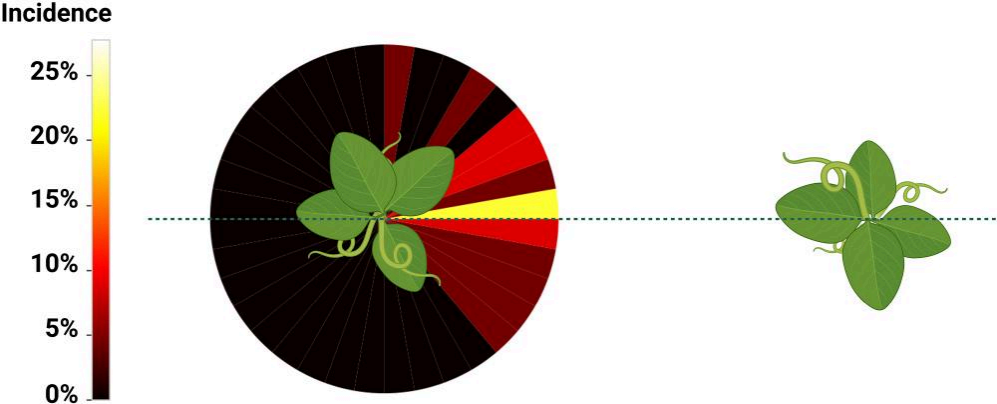
Graphical representation of the incidence of root growth angle $\theta_{g}$ that simulates the actual experimental condition with the study plant on the left and the neighbouring plant on the right. The circle around the plant reports the incidence of values for root growth angle $\theta_{g}$ in space. The colour code represents the actual range of values in that area according to the scale on the left.

If the root of the other plant becomes the goal of its aggregative behaviour, the direction of growth should be exactly towards the axis connecting the two plants, so that the angle between the direction of growth and the axis should be 0°. With this being a natural phenomenon, we expected that the collected values for this angle were distributed as a normal distribution centred around the value of 0°. We reported the incidence of $\theta_{g}$ values in a graphical representation that simulates the actual experimental condition with the neighbouring plant, as shown in Fig. 9.

From the graphical depiction of the data, the distribution seems to be close to normal around the zero value. To statistically test the normality, we applied the Shapiro–Wilk test (Shapiro and Wilk 1965) with a significance level (*α*) set to 0.05. The test statistic obtained was 0.9766, with a corresponding *P*-value of .9080. Given that the *P*-value exceeds the alpha threshold of 0.05, it fails to reject the null hypothesis of normality. This indicates there is insufficient evidence to conclude that the sample data significantly deviate from a normal distribution. Therefore, the sample data appear to be normally distributed with the 0.05 significance level. We performed a Shapiro–Wilk test to assess the normality of the distributions of the growth angle values for pea plants and maize plants separately. The *α* was set to 0.05. For pea plants, the test statistic obtained was 0.9099, with a corresponding *P*-value of .2431 that exceeds the alpha threshold of 0.05, failing to reject the null hypothesis of normality. For maize plants, the test statistic obtained was 0.9205, with a corresponding *P*-value of .4735 that also exceeds the alpha threshold of 0.05, failing to reject the null hypothesis of normality.

## Discussion

The aim of the present study was to leverage on a novel approach to unveil insights on the dynamic growth of roots in the presence of a neighbouring plant.

Our results for maize plants show that kinematic features and root plastic growth are modulated depending on the presence of a neighbouring plant. When exploring the feature importance analysis, the primary root of a maize plant growing with a neighbouring plant shows more and faster nutations, a more complex curvature with higher tortuosity, and a faster relative growth rate of the primary root. These results show that root kinematics and growth-related features are modulated when growing in the social condition. Such modifications in root behaviour may reflect a strategy to optimize spatial positioning and resource acquisition when growing with a neighbour. The increased tortuosity and dynamic movement of the primary root suggest a potential enhanced ability to navigate soil, which could be crucial for foraging efficiency. Furthermore, these changes may be driven by below-ground plant–plant signalling, potentially mediated by root exudates.

Our evidence of root growth modulation when growing with a neighbouring plant are in line with other studies showing evidence of roots exhibiting avoidance/aggregation responses to the presence of neighbours (Cahill and McNickle 2011). Our investigation goes one step further, providing quantitative 3D and time-related information on the modulation of circumnutations and oscillatory features of the root tip. Investigating circumnutations is of key relevance to understand their role in regulating exploratory behaviour during root growth: the helical movement of the root tip is associated with improved efficacy in substrate penetration. This aspect is critical for plant establishment and resources acquisition (Taylor et al. 2021).

To perform a first investigation on the effects of conspecific and heterospecific neighbours on the primary root movements of maize and pea plants, primary root movements were classified into three possible behaviours (i.e. aggregative, avoidant, and neutral). The evaluation of the incidence of these behaviours, while on a limited sample size, seem to provide an initial glimpse on the ability of maize and pea plants to alter their responses when growing close to a conspecific or heterospecific neighbour. When the neighbour is a conspecific, they tend to be more aggregative, while they tend to be more avoidant and neutral with a heterospecific neighbour. Our sample size limits the conclusiveness of these results, making them preliminary, but while preliminary, these findings are in line the idea that some species can alter their root responses according to the identity of the neighbour (Mahall and Callaway 1991, Bartelheimer et al. 2006, Callaway and Mahall 2007, Murphy and Dudley 2009, Fang et al. 2011, 2013). In accordance with Ciszak et al. (2012), our findings may be explained in the context of swarm behaviour whereby roots would coordinate their growth to optimally exploit soil resources (Baluška et al. 2010). Further investigation with a larger sample size is necessary to provide conclusive evidence. For a future comprehensive analysis of plant responses, it would be important to investigate plant response in relation to the response of the neighbour.

Most studies investigating plant root growth in social conditions focus on biomass allocation and static evaluation of the root architecture. Here, we propose a novel approach that examines the modulation of the kinematics and oscillatory features of the root tip in response to a neighbour. Present findings suggest that circumnutations of the primary root and their effective modulation during the first days of growth may provide a competitive advantage when growing with a neighbour. Faster circumnutations enhance the root tip’s ability of soil exploration and substrate penetration (Del Dottore et al. 2016, Yokawa and Baluška 2018, Taylor et al. 2021), thereby increasing the effectiveness of its movement.

We deepened this analysis by investigating whether evidence of directional movements towards a neighbouring root could be observed in maize and pea plants showing aggregative behaviour. Our findings suggest that roots seem to be able to detect neighbouring roots with a level of good geometrical precision and demonstrate the ability to initiate a directional movement towards them through plastic growth. These findings align with the idea that roots perceive exogenous environments and establish interactions with their elements by regulating and operating directional movements in advance.

It should be noted, however, that this study is not without limitations. Generalization of the responses found in this study requires the use of varying target species and varying competitive species. Although both the target and the neighbour species used in the study are model species, further studies with wild target and neighbour plants that co-occur in nature might be more representative of the kind of competitive interactions encountered by species in natural settings. Finally, in this study, we focused on the primary root as a proxy for early-stage exploratory behaviour. Future studies could also investigate the kinematics of secondary roots.

## Conclusion

This study presents a step towards a deeper understanding of the dynamic interactions between neighbouring roots during plant growth. By using a new approach able to provide 3D and time-related information on root growth, it provides preliminary results towards a more comprehensive understanding of below-ground interactions for co-cultivation and social growing. A deeper understanding of these responses provides valuable insights into plant adaptive strategies and could have implications for improving crop management and productivity in dense planting conditions. The hydroponic setup allowed the control and uniform distribution of nutrients, which was crucial to isolate the effect of the neighbouring plant and to develop specific and repeatable protocols for *in vivo* measurements with good time resolution. This enables the investigation of how plants cope with surrounding challenges and environmental changes that drive the readjustments of root growth and development. This study provides new insights into the effects of a neighbour on the primary root development during the first days of growth, showing that the presence of another plant has an impact on root growth and that these effects exhibit both temporal and spatial dynamics. It gives new insight into plant behaviours underlying survival chances in a competing environment where other plants have access to the same resources.

## Supplementary Material

plaf031_Supplementary_Data

## Data Availability

All the 3D trajectories of root tips used in this paper are available at https://zenodo.org/record/8422242 (individual condition) and at https://zenodo.org/records/14288939 (social conditions).
